# Differential Relationship between Intermuscular Adipose Depots with Indices of Cardiometabolic Health

**DOI:** 10.1155/2018/2751250

**Published:** 2018-09-04

**Authors:** Robert E. Bergia, Jung Eun Kim, Wayne W. Campbell

**Affiliations:** ^1^Department of Nutrition Science, Purdue University, West Lafayette IN 47907, USA; ^2^Food Science and Technology Programme, c/o Department of Chemistry, National University of Singapore, Singapore, Singapore 119077

## Abstract

**Background:**

Globally, accumulation of intermuscular adipose tissue (IMAT) is positively associated with insulin resistance. Whether this association is observed consistently in different skeletal muscles and encompasses other markers of cardiometabolic health is not well known.

**Objectives:**

The purpose of this secondary analysis study was to investigate associations among thigh or calf IMAT stores and indices of cardiometabolic health in adults who are overweight and obese participating in dietary interventions. A subset of calf data was analyzed to assess relations between IMAT in the gastrocnemius (type II fiber predominance) and soleus (type I fiber predominance) with markers of cardiometabolic health.

**Materials and Methods:**

Thigh and calf compositions were assessed via magnetic resonance imaging in 113 subjects (mean ± SD, age: 50 ± 16 y (range: 21–77 y), BMI: 31 ± 3 kg/m^2^), 103 of which completed dietary interventions with or without energy restriction-induced weight loss. A subset of data (*n* = 37) was analyzed for relations between muscle compartments (gastrocnemius and soleus) and cardiometabolic health. IMAT was regressed separately against fasting serum glucose concentrations, insulin, homeostatic model assessment-insulin resistance (HOMA-IR), and lipids and lipoproteins.

**Results:**

In general, total thigh IMAT was predictive of markers of glucose control, while total calf IMAT was not. Specifically, baseline thigh IMAT was positively associated with fasting glucose, insulin, and HOMA-IR. IMAT content changes in any depot did not predict improvement in cardiometabolic health.

**Conclusions:**

The strength of the relationship between IMAT and glucose control-related indices of cardiometabolic health is dependent on IMAT location. Specifically, greater IMAT in the thigh is a better predictor of cardiometabolic risk than greater IMAT in the calf in adults who are overweight and obese.

## 1. Introduction

Obesity is implicated in the development of metabolic syndrome, a multifaceted disorder encompassing insulin resistance, hypertension, and dyslipidemia [1]. While the general deleterious effects of greater adiposity are well documented, the concept that the metabolic consequences associated with obesity may be more related to regional body fat distribution and ectopic fat deposition as opposed to absolute quantity has more recently emerged [2, 3]. Visceral adipose tissue (VAT), one such depot of ectopic fat, is strongly associated with metabolic syndrome in spite of VATs' relatively small contribution to total adiposity [3–6]. Advances in imaging technology have enabled identification of intermuscular adipose tissue (IMAT; adipose tissue between muscle groups and beneath the fascia [7]), a unique ectopic adipose depot implicated in an array of pathological outcomes akin to VAT [8]. Until recently, IMAT has not garnered a level of attention commensurate to its potential impact on the metabolic profile.

Intermuscular adipose tissue is associated with greater risk of all-cause mortality; each one-standard deviation (SD) increase in IMAT (~6.8% greater IMAT) is associated with a 40% greater mortality risk over a 10-year period [9]. IMAT content is higher in individuals with obesity and type 2 diabetes [10–13]. While obesity and elevated body mass index (BMI) scores typically coincide with metabolic detriments, IMAT is independently linked with the metabolic syndrome in normal-weight and overweight men [14]. This suggests that there are metabolic consequences of IMAT accumulation separate from consequences of obesity.

Mounting evidence implicates both relative and absolute IMAT quantity to be consistently associated with insulin resistance [8, 10, 15, 16] and inconsistently associated with the worsened lipid-lipoprotein profile [6, 8, 17]. Determination of whole-body IMAT is a time-consuming and costly endeavor [16]. Often, sections of the lower limbs are analyzed by magnetic resonance imaging (MRI) or computed tomography to quantify relative IMAT content. These sections of either the thigh [15, 17–21] or calf [12, 13, 22, 23] are often interpreted as being representative of whole-body muscle composition. However, IMAT infiltration into skeletal muscle may be muscle- or muscle-compartment specific [23, 24]. This finding presents a serious obstacle when interpreting data from the body of literature on IMAT, which incorporates numerous different imaging techniques and extrapolates findings from different anatomical sites [25]. Additionally, difficulty exists in determining if IMAT directly affects metabolic function or is merely a marker of impairment [26]. This uncertainty arises from temporal issues such as IMAT showing strong associations with insulin resistance before interventions, yet failing to predict improvement in insulin sensitivity with reductions in IMAT [15, 27].

Given these shortcomings, the primary aim of the current research was to (1) investigate associations between thigh or calf IMAT stores and indices of cardiometabolic health with a special consideration of determining a potential differential predictive ability between anatomical sites analyzed. The secondary aims were to (2) analyze the relations between IMAT in the soleus (type I fiber predominance) or gastrocnemius (type II fiber predominance) and indices of cardiometabolic health and (3) investigate associations between longitudinal changes in each of the IMAT compartments and changes in cardiometabolic health parameters. We hypothesized that the greater IMAT content would be associated with worsened indices of cardiometabolic health with no difference on the basis of (1) location (thigh vs. calf) or (2) fiber type predominance (soleus vs. gastrocnemius). Further, we hypothesized that (3) IMAT reductions in any depot would not predict longitudinal improvement in indices of cardiometabolic health.

## 2. Materials and Methods

### 2.1. Subjects and Experimental Design

The current study involved retrospective analysis of baseline data from one cross-sectional study and baseline and postintervention data from three clinical studies (Table 1). The rationale for conducting this secondary analysis was to pool and analyze data from disparate research studies conducted by our research group to get a global view of how dietary interventions influence changes in IMAT and its relationship with indices of cardiometabolic health. As such, the clinical studies included dietary interventions with (*n* = 2) or without (*n* = 1) weight loss and with (*n* = 1) or without (*n* = 2) exercise. Study participants were overweight and obese males and females recruited from the greater Lafayette area in Indiana. Inclusion criteria consisted of the following: stable weight (±4.5 kg within the past 6 months), nonsmoking, no acute illness, and not clinically diagnosed with diabetes mellitus. Baseline data were used from 113 subjects (39 males and 74 females), with 10 subjects from a cross-sectional study supplementing 103 subjects from the clinical studies (pre- and postintervention data from 93 subjects). Data were extracted from original research files from parent studies and compiled by means of double entry. The Purdue University Biomedical Institutional Review Board approved the study protocols, and all subjects provided written informed consent and received monetary compensation for their participation. Clinical trial profiles of the four original studies can be found at clinicaltrials.gov under NCT01396915, NCT01692860, NCT02187965, and NCT02066948.

### 2.2. Anthropometric Measurements and Body Composition

Subjects' height (±0.1 cm) and weight (±0.1 kg) were measured using a wall-mounted stadiometer and a digital balance scale, respectively. These measurements were used to calculate BMI (kg/m^2^).

### 2.3. Magnetic Resonance Imaging and Image Analysis

MRI image acquisition and analysis were described previously [29]. Briefly, overnight fasted subjects arrived at a MRI facility (InnerVision West, West Lafayette, IN) and were scanned using a 3.0 T Signa HDx whole-body MRI machine (General Electric, Waukesha, WI). Prior to scanning, subjects were instructed to lie in a supine position on a MRI-safe bed for 1 hour to minimize the effects of body position on the scanning outcomes [30]. Following the rest period, subjects were shifted to the MRI machine bed while remaining in the supine position, and the MRI device captured images of the dominant leg. Twenty-seven image slices were obtained, and they were analyzed using Medical Image Processing, Analysis, and Visualization (MIPAV) MRI analysis software (v 7.0, Center for Information Technology, National Institutes of Health, Bethesda, MD) beginning with the first slice after the appearance of the *rectus femoris*, proceeding with every third slice, and ending with the appearance of the *gluteus maximus*. Total tissue, muscle tissue, subcutaneous adipose tissue (SAT), and IMAT regions were identified, and the respective areas were calculated. Each slice chosen for analysis represented three slices in total: itself, the previous slice, and the following slice. The average IMAT area (IMATa; absolute IMAT quantity, cm^2^), average muscle tissue area (MT, cm^2^), average subcutaneous adipose tissue (cm^2^), and average total cross-sectional area (CSA; cm^2^) were determined. IMAT, SAT, and MT in the total thigh and total calf were standardized to CSA. The gastrocnemius and soleus were semiautomatically segmented and analyzed individually. Average gastrocnemius IMAT and soleus IMAT areas were standardized to the average calf muscle area for analysis. MRI images from parent studies were reanalyzed with the aforementioned protocol to reconcile potential differences in imaging methodology among studies.

### 2.4. Blood Collection and Analysis

Following a 10 h overnight fast, blood samples were obtained from an antecubital vein and placed in tubes containing a clot activator to obtain serum or sodium heparin to obtain plasma. Serum tubes were held at room temperature for 30 minutes and then centrifuged at 4000xg at 4°C for 15 minutes. Serum tubes were sent to a commercial analytical laboratory (Mid America Clinical Laboratories, Indianapolis, IN) for the determination of concentrations of lipids and lipoproteins, including high-density lipoprotein cholesterol (HDL), low-density lipoprotein cholesterol (LDL), total cholesterol (TC), and triglycerides (TG). Serum or plasma glucose concentrations were measured using a photometric assay (Chemistry Immuno Analyzer AU5700, Olympus, Center Valley, PA). Serum insulin concentrations were measured in duplicate using an electrochemiluminescence immunoassay method on the Elecsys 2010 analyzer (Roche 108 Diagnostic Systems). The homeostatic model assessment of insulin resistance (HOMA-IR) was calculated as previously described [31].

### 2.5. Statistical Analysis

A multiple linear regression model was used to assess the associations of IMAT in each depot with indices of cardiometabolic health. Specifically, thigh IMAT or calf IMAT (standardized to CSA) and soleus IMAT or gastrocnemius IMAT (standardized to MT) were regressed against glucose, insulin, HOMA-IR, TG, TC, LDL, HDL, and TC : HDL. All estimates were adjusted for age and sex. Longitudinal estimates were adjusted for age, sex, and baseline-dependent variable. Multiple linear regression *P* values are presented raw and adjusted for multiple testing using the false discovery rate procedure [32]. Independent two-tailed *t*-tests were used to compare baseline subject characteristic data of males and females, and paired two-tailed *t*-tests were used to determine time effects. All analyses were performed using SAS 9.4 (SAS Institute Inc., Cary, NC, USA). Data are presented as mean ± standard deviation (SD), and adjusted regression coefficients (*β* ^∗^) are reported (statistical significance accepted at *P* < 0.05).

## 3. Results

### 3.1. Demographic, Clinical, and Muscle Composition Data

The characteristics of the 113 subjects (107 Caucasian, 4 African American, and 2 Asian) are presented in Table 2. With the exception of HDL, all indices of cardiometabolic health improved over time. There were no significant sex differences for age, BMI, LDL, TC, TG, insulin, and HOMA-IR (Supplemental Table 1). Overall, thigh and calf CSA and total IMATa decreased over time due to the interventions (Table 2). Reductions in SAT (thigh: −20.94 ± 13.58 cm^2^, calf: −3.31 ± 2.96 cm^2^) were greater than reductions in MT (thigh: −3.49 ± 6.92 cm^2^, calf: −2.43 ± 2.57 cm^2^), indicating improved body composition. Relative decreases in IMAT were greater than decreases in CSA.

### 3.2. Associations of Thigh or Calf IMAT with Cardiometabolic Health Indices

#### 3.2.1. Baseline Associations of Thigh or Calf IMAT with Cardiometabolic Health Indices

Greater relative thigh IMAT was associated with all measured markers of glucose control (Table 3). Specifically, thigh IMAT was associated with greater glucose concentrations, insulin, and HOMA-IR. Conversely, there were no relationships between calf IMAT and markers of glucose control.

While trends for greater relative thigh IMAT being associated with some, but not all measured lipid and lipoprotein outcomes, were apparent, the significance was lost with FDR adjustment for multiple testing (Table 3). There was no relationship between the calf IMAT and lipid-lipoprotein profile.

#### 3.2.2. Associations between Changes in Thigh or Calf IMAT and Changes in Cardiometabolic Health Indices

Thigh or calf IMAT reductions were not associated with changes in any index of cardiometabolic health (Table 3).

### 3.3. Associations of Calf Muscle Compartment IMAT with Cardiometabolic Health Indices

#### 3.3.1. Demographic, Clinical, and Muscle Composition Results

Supplemental Table 3 presents the characteristics of the 37 subjects in the calf muscle compartment analysis subgroup. Dietary interventions decreased IMAT within and the muscle area of the soleus and gastrocnemius. IMAT represented ~8.7% of the muscle area analyzed in the gastrocnemius preintervention and was reduced to 7.8% of the muscle area analyzed postintervention. There was a relatively greater IMAT content in the soleus, with 11.6% IMAT at preintervention, which was reduced to 10.7% postintervention. Absolute, but not relative, reductions of IMAT in the soleus were greater than those in the gastrocnemius.

#### 3.3.2. Baseline Associations between Gastrocnemius and Soleus IMAT and Cardiometabolic Health Indices

Neither gastrocnemius nor soleus IMAT was associated with any index of cardiometabolic health (Supplemental Table 4).

#### 3.3.3. Associations between Changes in Gastrocnemius and Soleus IMAT and Changes in Cardiometabolic Health Indices

Similar to total calf and thigh IMAT, changes in gastrocnemius and soleus IMAT did not predict improvement in markers of any index of cardiometabolic health (Supplemental Table 4).

## 4. Discussion

Contrary to our hypothesis that greater IMAT would be associated with worsened cardiometabolic health with no difference on the basis of location or fiber type predominance, our results suggest that (1) IMAT in the thigh was more predictive of cardiometabolic dysfunction than IMAT in the calf. Consistent with our hypothesis, there was no differential relationship between soleus and gastrocnemius IMAT and indices of cardiometabolic health. Also consistent with our hypothesis and those of other research studies, (3) changes in IMAT did not reliably predict improvements in indices of cardiometabolic health [15, 19, 27, 33].

Understanding of adipose tissues has progressed from the notion that these tissues were inert storage depots to our current understanding of adipose as an important endocrine organ [34]. As part of this evolution, the concept that not all adipose tissues possess the same biochemical attributes and confer similar metabolic risk was recognized [34–36]. Investigations of associations between body fat distribution and metabolic health include comparing subcutaneous adipose tissue and VAT [10, 14]. VAT now has a well-defined role in increasing cardiometabolic risk factors relative to subcutaneous adipose tissue [36]. In a similar manner, the comparison of different ectopic fat depots is not without precedent. There is some debate over whether IMAT contributes to metabolic disturbance in a similar fashion to VAT. Previously, the relative importance of VAT and IMAT on the metabolic profile was compared [8, 16]. In some research studies, VAT appeared to possess stronger relations with indices of cardiometabolic health [6, 20], while IMAT was a better predictor of cardiometabolic health in other research studies [8, 17]. Akin to comparisons conducted between VAT and IMAT, various IMAT depots were compared. Investigators compared thigh and calf muscle composition and concluded that thigh muscle quality (reduced IMAT) was the stronger contributor to physical function [37]. Similarly, we now conclude that thigh IMAT is the stronger contributor to cardiometabolic dysfunction. Our findings agree with other research studies that significant relations exist between IMAT in the thigh and glucose [8, 13, 21, 38], insulin [21, 39], and HOMA-IR [6, 39]. While mechanistic support is lacking and some research studies do not support the relation between IMAT and markers of glucose control [33, 40], our results support the preponderance of research suggesting that IMAT contributes to (or is a product of) disrupted glucose homeostasis [26].

While there is strong evidence supporting the relationship between greater IMAT content and impaired glucose homeostasis, there is considerably less support in favor of relations between IMAT and cardiovascular disease risk factors. Relations between IMAT and TC [8] and TG [6] and an inverse association with HDL [6] were reported. However, null findings concerning the relations between IMAT and TC [6, 18, 38], TG [8, 18, 38], LDL [6, 18], and HDL [8, 38] are more frequently reported. Consistent with this, we did not detect any associations between IMAT and lipid-lipoprotein profile in the overall sample. Discordance in the literature towards lipid findings may relate to differences in subject characteristics. One study found that loss of IMAT was associated with increases in HDL and LDL particle size and a subsequent decrease in cardiovascular disease risk in men, but not in women [17]. Our findings are not consistent with these results, as we detected a stronger association between IMAT and lipid-lipoprotein profile in women. Specifically, our results indicate that IMAT in the thigh was associated with elevated TG, TC, LDL, and TC : HDL in women, but not in men. These results should be interpreted with caution, however, as there were almost twice as many women as men in the analysis. Thus, we cannot rule out the fact of potentially being underpowered to detect these associations in the male subgroup. Previously, investigators reported that the relationship between IMAT and total cholesterol was markedly stronger in Caucasians than in African Americans [8]. The impact of IMAT on the lipid-lipoprotein profile may be underestimated due to the purposeful oversampling of African Americans in some of the most influential IMAT research parent studies [8, 13, 17, 38, 41]. Our disparate findings regarding IMAT and cardiovascular risk factors may be at odds with the majority of the literature because our study population was predominantly Caucasian.

In regard to the longitudinal findings, it is important to note that our results were independent of overall adiposity changes as a result of the dietary interventions, as changes in body size were accounted for with the standardization of IMAT to CSA. With that said, changes in IMAT typically do not predict improvement in risk factors for cardiometabolic disease [15, 27]. Our findings in regard to the relations between changes in segmental IMAT and changes in cardiometabolic health indices support this consensus.

The current observational research did not include means of delineating mechanisms of IMAT accumulation and its contribution to worsened cardiometabolic health. Myogenic cells possess the ability to differentiate into adipocytes [42], and the relative conversion rate is potentially modifiable through diet, exercise, and pharmacological means [43, 44]. Hyperglycemia and elevated concentrations of long-chain fatty acids increase adipogenic conversion from muscle stem cells [43, 44]. In a bidirectional fashion, just as disturbances to metabolic health could lead to increases in IMAT, increases in IMAT can feedback and contribute to alterations in metabolic health. As muscle is the primary site for glucose metabolism, the close proximity of IMAT to muscle can act in a manner analogous to VAT and the liver by altering the local metabolic environment [8]. IMAT is thought to exert its deleterious effects primarily through impairing insulin action and glucose metabolism [45, 46]. IMAT can also contribute to the development of insulin resistance by impairing blood flow to muscles [15], inducing a proinflammatory environment in muscle [47, 48] and increasing oxidative stress [49]. Due to the interconnections between insulin resistance, inflammation, and dyslipidemia, these potential IMAT-induced alterations can impact cardiovascular health parameters as well [1]. Despite these associations and supporting data, a fully realized model in which IMAT definitively causes worsened metabolic health is yet to be widely accepted.

Our use of MRI to quantify IMAT is a strength of the current research. MRI allows researchers to directly measure IMAT [50] and possesses greater sensitivity than computed tomography [51], which indirectly measures IMAT. Pooled analysis of three RCTs from our research group make this the largest investigation of intervention-induced changes in IMAT on cardiometabolic health indices, to the authors' knowledge. This research is not without limitation. We recognize that dissimilar intervention features, particularly the presence or absence of exercise training and energy restriction, in secondary analysis of RCTs are significant sources of heterogeneity and may limit the ability to detect meaningful associations. Therefore, we want to stress our objective to obtain a global view of how dietary interventions influence changes in IMAT; these findings are exploratory in nature. Further, the lack of clear mechanistic support hinders conclusions we are able to draw from the current research.

## 5. Conclusions

In conclusion, our findings suggest that relations between IMAT depots and indices of cardiometabolic health vary by body sites. Specifically, greater IMAT in the thigh is a better predictor of cardiometabolic risk than greater IMAT in the calf. Consistent with other research studies, changes in thigh or calf IMAT do not reliably predict improvements in cardiometabolic health parameters.

## Figures and Tables

**Table 1:** Descriptions of the randomized controlled trials included in a secondary analysis on the relationship between intermuscular adipose tissue and indices of cardiometabolic health^¥^.

Author (year)	Sample size	Duration (wk)	Age (y)	Energy restriction	Exercise training
Campbell et al. (unpublished)	10	CS	70 ± 4	N/A	N/A
Hudson et al. (2017) [28]	38	16	34 ± 9	750 kcal/d ER	RT—3x/wk
Wright et al. (unpublished)	19	12	70 ± 4	No ER	No training
Zhou et al. (unpublished)	46	16	52 ± 8	750 kcal/d ER	No training

^¥^Individual study details regarding study characteristics (duration, energy restriction component, and exercise training component); CS: cross-sectional; ER: energy restriction; RT: resistance training. Data are presented as means ± SD, where appropriate.

**Table 2:** Clinical and cardiometabolic characteristics of all subjects.

General characteristics	*n* = 113 (74 F, 39 M)		
Age (y)	50 ± 15		
Height (cm)	170 ± 10		
Weight (kg)	90.1 ± 13.3		
BMI (kg/m^2^)	31.2 ± 2.9		
Cardiometabolic health	Pre	Post	Change
Glucose (mmol/l)	5.16 ± 0.49	5.02 ± 8.8	**−0.12 ± 7.8** ^∗^
Insulin (pmol/l)	84.73 ± 44.45	55.56 ± 28.47	**−27.78 ± 40.98** ^∗^
HOMA-IR	2.85 ± 1.64	1.82 ± 1.01	**−0.97 ± 1.47** ^∗^
Total cholesterol (mmol/l)	10.46 ± 1.97	9.30 ± 1.82	**−0.99 ± 1.24** ^∗^
Triglycerides (mmol/l)	6.79 ± 2.89	5.55 ± 2.31	**−1.39 ± 2.54** ^∗^
HDL (mmol/l)	2.64 ± 0.84	2.50 ± 0.61	0.01 ± 0.36
LDL (mmol/l)	6.47 ± 1.74	5.68 ± 1.61	**−0.74 ± 0.96** ^∗^
TC : HDL	0.24 ± 1.34	0.22 ± 1.06	**−0.03 ± 0.75** ^∗^
Thigh IMAT			
CSA (cm^2^)^¥^	229.48 ± 47.09	212.24 ± 40.31	**−24.46 ± 16.10** ^∗^
IMATa (cm^2^)	11.12 ± 3.46	9.84 ± 3.10	**−1.46 ± 1.01** ^∗^
IMAT	0.0498 ± 0.0164	0.0478 ± 0.0159	**−0.0015 ± 0.0044** ^∗^
MT (cm^2^)^¥^	115.55 ± 32.14	115.11 ± 30.27	**−3.49 ± 6.92** ^∗^
SAT (cm^2^)	113.71 ± 49.18	97.13 ± 42.04	**−20.94 ± 13.58** ^∗^
Calf IMAT			
CSA (cm^2^)^¥^	97.68 ± 15.31	92.47 ± 14.22	**−5.74 ± 4.64** ^∗^
IMATa (cm^2^)	6.49 ± 2.31	5.96 ± 2.29	**−0.63 ± 0.83** ^∗^
IMAT	0.0670 ± 0.0229	0.0649 ± 0.0238	**−0.0027 ± 0.0073** ^∗^
MT (cm^2^)^¥^	61.09 ± 13.26	58.67 ± 11.68	**−2.43 ± 2.67** ^∗^
SAT (cm^2^)	36.59 ± 15.29	33.44 ± 13.91	**−3.31 ± 2.96** ^∗^

Data are mean ± SD; significance determined through paired *t*-tests (^∗^ *P* values < 0.05). CSA: cross-sectional area of the segment; HDL: high-density lipoprotein; HOMA-IR: homeostatic model assessment of insulin resistance; IMAT: intermuscular adipose tissue (standardized to cross-sectional area); IMATa: intermuscular adipose tissue (absolute quantity); LDL: low-density lipoprotein; MT: muscle tissue; SAT: subcutaneous adipose tissue; TC: total cholesterol; TG: triglyceride. ^¥^Bone area removed.

**(a):** 

Baseline associations	Thigh IMAT : CSA (*n* = 108)		Calf IMAT : CSA (*n* = 95)	
	*β* ^a^ (95% CI)	*P* value (FDR-adjusted *P*)	*β* ^a^ (95% CI)	*P* value (FDR-adjusted *P*)
Glucose (mmol/l)	6.38 (1.17, 11.59)	**0.020** ^∗^ ** (0.041)**	−0.69 (−5.20, 3.81)	0.761 (0.854)
Insulin (pmol/l)	886.24 (312.43, 1460.06)	**0.009** ^∗^ ** (0.026)**	128.13 (−380.63, 636.90)	0.618 (0.854)
HOMA-IR	32.77 (11.64, 53.90)	**0.009** ^∗^ ** (0.026)**	1.74 (−17.04, 20.51)	0.854 (0.854)
Triglycerides (mmol/l)	47.33 (15.21, 79.44)	0.079 (0.349)	−2.92 (−33.41, 27.57)	0.849 (0.849)
Total cholesterol (mmol/l)	19.67 (−3.19, 42.54)	0.155 (0.349)	11.14 (−9.18, 31.46)	0.279 (0.349)
LDL (mmol/l)	13.59 (−7.45, 34.62)	0.257 (0.349)	10.60 (−8.08, 29.29)	0.263 (0.349)
HDL (mmol/l)	−7.58 (−16.42, 1.26)	0.203 (0.349)	−2.66 (−8.97, 3.66)	0.405 (0.450)
TC:HDL	21.85 (7.46, 36.24)	0.033 (0.332)	9.84 (−3.62, 23.31)	0.150 (0.349)

**(b):** 

∆Associations	∆Thigh IMAT : CSA (*n* = 94)		∆Calf IMAT : CSA (*n* = 90)	
	*β* ^a^ (95% CI)	*P* value (FDR-adjusted *P*)	*β* ^a^ (95% CI)	*P* value (FDR-adjusted *P*)
**∆**Glucose (mmol/l)	−8.22 (−20.73, 4.28)	0.197 (0.421)	4.95 (−8.94, 18.84)	0.480 (0.721)
**∆**Insulin (pmol/l)	−437.86 (−1908.87, 1033.14)	0.883 (0.883)	968.92 (−359.38, 2297.22)	0.151 (0.421)
**∆**HOMA-IR	−15.37 (−67.12, 36.37)	0.868 (0.883)	30.19 (−17.42, 77.80)	0.149 (0.421)
**∆**Triglycerides (mmol/l)	−34.65 (−117.29, 47.98)	0.677 (0.752)	26.35 (−53.60, 106.31)	0.514 (0.752)
**∆**Total cholesterol (mmol/l)	−7.30 (−48.43, 33.83)	0.560 (0.752)	14.15 (−26.22, 54.52)	0.488 (0.752)
**∆**LDL (mmol/l)	6.00 (−26.71, 38.71)	0.949 (0.949)	16.90 (−14.62, 48.42)	0.289 (0.752)
**∆**HDL (mmol/l)	−1.74 (−13.65, 10.18)	0.578 (0.752)	−5.10 (−16.82, 6.62)	0.390 (0.752)
**∆**TC : HDL	−0.51 (−24.84, 23.83)	0.647 (0.752)	17.50 (−6.38, 41.39)	0.149 (0.752)

All estimates are adjusted for age and sex. Longitudinal analyses adjusted for age and baseline-dependent variable. ^a^Estimates of the adjusted regression coefficient between glucose, insulin, HOMA-IR, TC, TG, LDL, HDL, and TC : HDL with thigh and calf IMAT; ^∗^ *P* values < 0.05. CI: confidence interval; HDL: high-density lipoprotein; HOMA-IR: homeostatic model assessment of insulin resistance; IMAT: intermuscular adipose tissue (standardized to cross-sectional area of the segment); LDL: low-density lipoprotein; TC: total cholesterol; TG: triglyceride.

## Data Availability

The data used to support the findings of this study are available from the corresponding author upon request.

## Supplementary Materials

Supplementary Materials The following Supporting Information is available through the online version of this article at the publisher's website. Table S1: clinical and cardiometabolic characteristics of all subjects by sex. Table S2: associations between thigh or calf IMAT with indices of cardiometabolic health by sex. Table S3: clinical and cardiometabolic characteristics of the calf composition subgroup by sex. Table S4: associations between gastrocnemius and soleus IMAT with indices of cardiometabolic health.
