# Supplementary material for: Differential Relationship between Intermuscular Adipose Depots with Indices of Cardiometabolic Health
Source: Int J Endocrinol. 2018 Sep 4;2018:2751250. doi: 10.1155/2018/2751250 (PMC6142737; doi:10.1155/2018/2751250)
Supplement: Supplementary Materials — The following Supporting Information is available through the online version of this article at the publisher's website. Table S1: clinical and cardiometabolic characteristics of all subjects by sex. Table S2: associations between thigh or calf IMAT with indices of cardiometabolic health by sex. Table S3: clinical and cardiometabolic characteristics of the calf composition subgroup by sex. Table S4: associations between gastrocnemius and soleus IMAT with indices of cardiometabolic health. [file 2751250.f1.docx]

| **Supplemental Table 1.**  Clinical and cardiometabolic characteristics of all subjects by sex. | | | | | | | | |
| --- | --- | --- | --- | --- | --- | --- | --- | --- |
| **Outcome Variable** | | **Female**  ***(n = 74)*** | | | **Male**  ***(n = 39)*** | | | **P-Value**  *Group* |
| Age (yr) | | 50 ± 15 | | | 50 ± 17 | | | 0.966 |
| Height (cm) | | 164.54 ± 6.80 | | | 178.61 ± 7.36 | | | <0.001 |
| Weight (kg) | | 84.9 ± 10.4 | | | 99.9 ± 12.8 | | | <0.001 |
| BMI (kg/m^2^) | | 31.10 ± 2.90 | | | 31.28 ± 2.99 | | | 0.770 |
| **Cardiometabolic Health** | | *Pre-* | *Post-* | *Change* | *Pre-* | *Post-* | *Change* | *Group*time* |
|  | Glucose (mmol/l) | 5.04 ± 0.46^a^ | 4.95 ± 0.50 | -0.06 ± 0.44 | 5.38 ± 0.48^a^ | 5.16 ± 0.44 | **-0.22 ± 0.40*** | 0.084 |
|  | Insulin (pmol/l) | 81.95 ± 39.59 | 56.25 ± 28.47 | **-23.61 ± 41.67*** | 90.98 ± 51.39 | 54.87 ± 29.17 | **-35.42 ± 39.59*** | 0.203 |
|  | HOMA-IR | 2.66 ± 1.41 | 1.80 ± 0.96 | **-0.78 ± 1.45*** | 3.19 ± 1.97 | 1.86 ± 1.10 | **-1.32 ± 1.46*** | 0.092 |
|  | Total Cholesterol (mmol/l) | 10.56 ± 1.77 | 9.47 ± 1.87 | **-0.93 ± 1.09*** | 10.26 ± 2.29 | 8.98 ± 1.71 | **-1.11 ± 1.49*** | 0.516 |
|  | Triglycerides (mmol/l) | 6.28 ± 5.73^a^ | 5.36 ± 2.01 | **-1.03 ± 1.91*** | 7.74 ± 3.51^a^ | 5.90 ± 2.76 | **-2.03 ± 3.36*** | 0.106 |
|  | HDL (mmol/l) | 2.86 ± 0.86^a^ | 2.64 ± 0.54 | -0.03 ± 0.36 | 2.23 ± 0.64^a^ | 2.25 ± 0.66 | 0.09 ± 0.37 | 0.121 |
|  | LDL (mmol/l) | 6.44 ± 1.60 | 5.75 ± 1.67 | **-0.69 ± 0.99*** | 6.54 ± 1.99 | 5.56 ± 1.51 | **-0.84 ± 0.99*** | 0.463 |
|  | TC:HDL | 3.96 ± 1.20^a^ | 3.71 ± 0.96 | **-0.38 ± 0.66*** | 4.90 ± 1.39 ^a^ | 4.22 ± 1.17 | **-0.72 ± 0.87*** | 0.053 |
| **Thigh IMAT** | |  |  |  |  |  |  |  |
|  | CSA (cm²)^¥^ | 233.93 ± 47.06 | 214.84 ± 39.80 | **-26.41 ± 16.86*** | 221.3 ±46.64 | 207.58± 41.39 | **-20.91 ± 14.16*** | 0.115 |
|  | IMATa (cm²) | 10.75 ± 3.51 ^a^ | 9.46 ± 2.57 | **-1.30 ± 0.96*** | 12.37 ± 4.57^a^ | 10.92 ± 3.37 | **-2.27 ± 2.99*** | 0.076 |
|  | IMAT | 0.0469 ± 0.015 ^a^ | 0.0448 ± 0.013 | -0.0004 ± 0.004 | 0.0574 ± 0.022 ^a^ | 0.0537 ± 0.018 | **-0.006± 0.01*** | **0.026*** |
|  | MT (cm²)^¥^ | 97.99 ± 18.09 ^a^ | 98.71 ± 19.23 | **-1.97 ± 5.49*** | 147.90 ± 26.81 ^a^ | 144.54 ± 23.41 | **-6.27 ± 8.35*** | **0.011*** |
|  | SAT (cm²) | 135.28 ± 42.30 ^a^ | 116.13 ± 34.66 | **-24.36 ± 14.32*** | 73.40 ± 33.38 ^a^ | 63.04 ± 31.38 | **-14.63 ± 9.39*** | **<0.001*** |
| **Calf IMAT** | |  |  |  |  |  |  |  |
|  | CSA (cm²)^¥^ | 97.82± 15.51 | 92.28 ± 14.14 | **-5.614 ± 4.363*** | 97.42 ± 15.15 | 92.83 ± 14.60 | **-5.99 ± 5.22*** | 0.719 |
|  | IMATa (cm²) | 5.80 ± 1.79 ^a^ | 5.29 ± 1.66 | **-0.51 ± 0.53*** | 7.80 ± 2.62 ^a^ | 7.21 ± 2.77 | **-0.867 ± 1.205*** | 0.134 |
|  | IMAT | 0.0599 ± 0.02 ^a^ | 0.0578 ± 0.02 | **-0.0021± 0.005*** | 0.0802 ± 0.025 ^a^ | 0.0781 ± 0.029 | **-0.0039 ± 0.01*** | 0.382 |
|  | MT (cm²)^¥^ | 54.22 ± 8.71 ^a^ | 52.74 ± 8.40 | **-1.71 ± 1.86*** | 74.00 ± 10.41 ^a^ | 69.78 ± 8.41 | **-3.87 ± 3.42*** | **0.003*** |
|  | SAT (cm²) | 43.60 ± 12.93 ^a^ | 38.89 ± 12.72 | **-3.90 ± 3.06*** | 23.42 ± 9.70 ^a^ | 23.05 ± 9.56 | **-2.12 ± 2.37*** | **0.006*** |
|  | Data are mean ± SD; significance determined through paired and independent T-Tests, P-values < .05 *  CSA, cross-sectional area of segment; HDL, high-density lipoprotein; HOMA:IR, homeostatic model assessment of insulin resistance; IMAT, intermuscular adipose tissue (standardized to cross-sectional area) IMATa, intermuscular adipose tissue (absolute quantity); LDL, low-density lipoprotein; MT; muscle tissue; SAT, subcutaneous adipose tissue; TC, total cholesterol; TG, triglyceride  ^a^Differences between males and females (P < 0.05)  ^¥^Bone area removed | | | | | | | |

| **Supplemental Table 2.**  Associations between thigh or calf IMAT with indices of cardiometabolic health by sex | | | | | | | | | |
| --- | --- | --- | --- | --- | --- | --- | --- | --- | --- |
| **Baseline Associations** | | **Female (n=74)** | | | | **Male (n=39)** | | | |
|  |  | **Thigh IMAT:CSA** | | **Calf IMAT:CSA** | | **Thigh IMAT:CSA** | | **Calf IMAT:CSA** | |
|  |  | **β ^a^ (95% CI)** | ***P* value** | **β ^a^ (95% CI)** | ***P* value** | **β ^a^ (95% CI)** | ***P* value** | **β ^a^ (95% CI)** | ***P* value** |
| Glucose (mmol/l) | | 6.84 (-0.72, 14.41) | **0.033*** | 1.30 (-5.02, 7.62) | 0.546 | 4.51 (-3.39, 12.41) | **0.004*** | -4.04 (-10.41, 2.32) | 0.775 |
| Insulin (pmol/l) | | 908.41 (13.89, 1802.92) | 0.054 | 137.51(-514.62, 789.65) | 0.797 | 925.77(-24.31,1876.54) | 0.083 | 70.14(-810.48, 950.08) | 0.872 |
| HOMA-IR | | 31.02 (-1.00, 63.04) | 0.063 | 3.43 (-19.80, 26.66) | 0.868 | 33.46 (-2.74, 69.66) | 0.045 | -2.40 (-35.65, 30.85) | 0.889 |
| Triglycerides (mmol/l) | | 42.27 (4.57, 79.98) | **0.042*** | 17.53 (-18.34, 53.40) | 0.341 | 72.16 (9.78, 134.54) | 0.307 | -23.46 (-80.96, 34.05) | 0.286 |
| Total Cholesterol (mmol/l) | | 37.74 (12.09, 63.39) | **<0.001*** | 27.79 (4.03, 51.56) | **0.009*** | 18.78 (-25.14, 62.71) | 0.705 | -1.19 (-37.14, 34.76) | 0.604 |
| LDL (mmol/l) | | 34.79 (10.21, 59.37) | **0.002*** | 30.20 (8.29, 52.28) | **0.004*** | 2.48 (-37.37, 43.43) | 0.903 | -6.86 (-39.42, 25.71) | 0.354 |
| HDL (mmol/l) | | -5.27 (-18.61, 8.07) | 0.989 | -5.89 (-14.61, 2.83) | 0.269 | -9.39 (-21.41, 2.62) | 0.766 | -0.27 (-1.57, 1.02) | 0.408 |
| TC:HDL | | 26.81 (8.30, 45.32) | **0.011*** | 24.43 (8.05, 40.81) | **0.004*** | 26.82 (1.12, 52.52) | 0.841 | -4.91 (-28.21, 18.40) | 0.271 |
| **Longitudinal Associations** | | **∆Thigh IMAT:CSA** | | **∆Calf IMAT:CSA** | | **∆Thigh IMAT:CSA** | | **∆Calf IMAT:CSA** | |
|  |  | **β ^a^ (95% CI)** | ***P* value** | **β ^a^ (95% CI)** | ***P* value** | **β ^a^ (95% CI)** | ***P* value** | **β ^a^ (95% CI)** | ***P* value** |
| **∆**Glucose (mmol/l) | | 8.29 (-23.36, 39.94) | 0.884 | 14.80 (-9.72, 39.31) | 0.058 | -13.09 (-30.90, 4.72) | 0.335 | 1.85 (-15.27, 18.96) | 0.888 |
| **∆**Insulin (pmol/l) | | -3388.47 (-7313.09, 536.15) | 0.260 | 1080.64 (-1287.60, 3448.19) | 0.338 | 96.54 (-1643.88, 1836.26) | 0.910 | 679.92 (-118.15, 2477.28) | 0.443 |
| **∆**HOMA-IR | | -108.01 (-243.15, 27.13) | 0.115 | 42.54 (-40.28, 125.35) | 0.263 | -0.54 (-66.10, 65.01) | 0.621 | 20.84 (-47.17, 88.85) | 0.480 |
| **∆**Triglycerides (mmol/l) | | -151.37 (-313.61, 10.88) | 0.275 | 21.64 (-80.96, 124.24) | 0.623 | 26.08 (-116.51, 168.68) | 0.634 | -0.21 (148.96, 148.56) | 0.998 |
| **∆**Total Cholesterol (mmol/l) | | -57.17 (-154.62, 40.28) | 0.434 | 9.79 (-52.23, 71.82) | 0.676 | 0.58 (-62.28, 63.44) | 0.869 | 4.28 (-6.52, 70.64) | 0.474 |
| **∆**LDL (mmol/l) | | -28.38 (-114.42, 57.66) | 0.601 | 14.71 (-39.19, 68.62) | 0.550 | 19.09 (-26.52, 64.69) | 0.399 | 14.48 (-30.84, 59.81) | 0.517 |
| **∆**HDL (mmol/l) | | 3.17 (-27.57, 33.89) | 0.843 | -9.50 (-29.34, 10.34) | 0.399 | -12.86 (-29.04, 3.33) | 0.906 | -2.84 (-19.89, 14.21) | 0.634 |
| **∆**TC:HDL | | -50.41 (-102.88, 2.07) | 0.059 | 18.15 (-17.24, 53.53) | 0.244 | 29.64 (-9.95, 69.22) | 0.711 | 13.05 (-27.22, 53.31) | 0.664 |
|  | All estimates are adjusted for age. Longitudinal analyses adjusted for age and baseline dependent variable.  ^a^Estimates of adjusted regression coefficient between glucose, insulin, HOMA-IR, TG, TC, LDL, HDL, and TC:HDL with thigh and calf IMAT; P-values < .05 *  CI, confidence interval; HDL, high-density lipoprotein; HOMA-IR, homeostatic model assessment of insulin resistance; IMAT, intermuscular adipose tissue (standardized to cross-sectional area of segment); LDL, low-density lipoprotein; TC, total cholesterol; TG, triglyceride | | | | | | | | |

| **Supplemental Table 3.** Clinical and cardiometabolic profile of calf composition subgroup by sex. | | | | |
| --- | --- | --- | --- | --- |
| Outcome Variable | | Women | Men | P-Value |
| **General Characteristics** | | *(n = 24)* | *(n = 13)* |  |
|  | **Age (yr)** | 36 ± 9 | 31 ± 9 | 0.146 |
|  | **Height (cm)** | 167.8 ± 6.1 | 178.2 ± 5.3 | **<0.001*** |
|  | **Weight (kg)** | 87.0 ± 9.8 | 99.9 ± 9.9 | **<0.001*** |
|  | **BMI (kg/m²)** | 30.8 ± 2.3 | 31.5 ± 2.6 | 0.456 |
| **Cardiometabolic Health** | |  |  |  |
|  | **Glucose (mmol/l)** | 5.00 ± 0.44 | 5.11 ± 0.28 | 0.426 |
|  | **Insulin (pmol/l)** | 97.23 ± 41.67 | 83.34 ± 55.56 | 0.364 |
|  | **HOMA-IR** | 3.05 ± 1.31 | 2.61 ± 1.70 | 0.405 |
|  | **Total Cholesterol (mmol/l)** | 10.33 ± 1.44 | 11.22 ± 2.44 | 0.230 |
|  | **Triglycerides (mmol/l)** | 6.39 ± 2.22 | 8.40 ± 3.94 | 0.101 |
|  | **HDL (mmol/l)** | 2.72 ± 0.56 | 2.06 ± 0.39 | **<0.001*** |
|  | **LDL (mmol/l)** | 6.33 ± 1.39 | 7.50 ± 2.11 | 0.049 |
|  | **TC:HDL** | 3.98 ± 1.12 | 5.62 ± 1.38 | **<0.001*** |
| Data are mean ± SD; significance determined through Independent T-Tests, P-values < .05 *  HDL, high-density lipoprotein; HOMA-IR, homeostatic model assessment of insulin resistance; LDL, low-density lipoprotein; TC, total cholesterol; TG, triglyceride | | | | |

| **Supplemental Table 4.** Associations between Gastrocnemius and Soleus IMAT with Indices of Cardiometabolic Health. | | | | |
| --- | --- | --- | --- | --- |
| **Baseline Associations** | **Gastrocnemius IMAT (n=37)** | | **Soleus IMAT (n=37)** | |
|  | **β ^a^ (95% CI)** | ***P*-value**  **(FDR-Adjusted P)** | **β ^a^ (95% CI)** | ***P*-value**  **(FDR-Adjusted P)** |
| Glucose (mmol/l) | -3.18 (-7.42, 1.05) | 0.136 (0.407) | -1.39 (-3.18, 0.41) | 0.125 (0.407) |
| Insulin (pmol/l) | 111.81 (-413.92, 637.55) | 0.666 (0.962) | 29.17 (-179.18, 236.82) | 0.777 (0.962) |
| HOMA-IR | 1.01 (-16.58, 18.59) | 0.908 (0.962) | -0.16(-7.11, 6.79) | 0.962 (0.962) |
| Triglycerides (mmol/l) | -10.97 (-41.37, 19.42) | 0.468 (0.932) | -4.10 (-17.04, 8.84) | 0.524 (0.932) |
| Total Cholesterol (mmol/l) | -8.62 (-27.77, 10.52) | 0.366 (0.932) | .35 (-7.88, 8.58) | 0.932 (0.932) |
| LDL (mmol/l) | -6.91 (-24.19, 10.38) | 0.422 (0.932) | 1.44 (-5.96, 8.84) | 0.694 (0.932) |
| HDL (mmol/l) | 0.52 (-4.63, 5.67) | 0.840 (0.932) | -0.27 (-2.46, 1.92) | 0.804 (0.932) |
| TC:HDL | -4.39 (-17.37, 8.58) | 0.496 (0.932) | 0.55 (-4.99, 6.10) | 0.840 (0.932) |
| **∆ Associations** | **∆ Gastrocnemius IMAT (n=37)** | | **∆ Soleus IMAT (n=37)** | |
|  | **β ^a^ (95% CI)** | ***P*-value**  **(FDR-Adjusted P)** | **β ^a^ (95% CI)** | ***P*-value**  **(FDR-Adjusted P)** |
| **∆**Glucose (mmol/l) | -3.18 (-20.37, 13.99) | 0.707 (0.942) | -3.82 (-12.69, 5.06) | 0.386 (0.942) |
| **∆**Insulin (pmol/l) | -125.01 (-2304.35, 2053.64) | 0.906 (0.942) | 276.41 (-861.87, 1415.39) | 0.619 (0.942) |
| **∆**HOMA-IR | -2.61 (-75.60, 70.38) | 0.942 (0.942) | 7.01 (-31.09, 45.11) | 0.706 (0.942) |
| **∆**Triglycerides (mmol/l) | 28.16 (-79.59, 135.92) | 0.597 (0.663) | 31.49 (-20.73, 83.72) | 0.227 (0.383) |
| **∆**Total Cholesterol (mmol/l) | 29.27 (-23.80, 82.33) | 0.268 (0.383) | 32.54 (8.28, 56.81) | 0.010 (0.104) |
| **∆**LDL (mmol/l) | 25.01 (-16.81, 66.82) | 0.231 (0.383) | 22.99 (3.07, 42.92) | 0.025 (0.127) |
| **∆**HDL (mmol/l) | -1.06 (-16.29, 14.18) | 0.888 (0.888) | 3.37 (-4.16, 10.90) | 0.367 (0.459) |
| **∆**TC:HDL | 22.41 (-4.12, 48.95) | 0.095 (0.932) | 12.85 (-0.16, 25.85) | 0.053 (0.175) |
| All estimates are adjusted for age and sex.  ^a^Estimates of adjusted regression coefficient between glucose, insulin, HOMA-IR, TG, TC, LDL, HDL, and TC:HDL with thigh and calf IMAT; P-values < .05 *  CI, confidence interval; HDL, high-density lipoprotein; HOMA:IR, homeostatic model assessment of insulin resistance; LDL, low-density lipoprotein; TC, total cholesterol; TG, triglyceride | | | | |
